# Longitudinal MRI changes after focal therapy for prostate cancer: cryotherapy vs. microwave tissue coagulation

**DOI:** 10.1007/s11604-025-01831-4

**Published:** 2025-07-10

**Authors:** Nana Kozawa, Kaori Yamada, Bunta Tokuda, Akiko Takahata, Yayoi Iwami, Toshiko Ito-Ihara, Atsuko Fujihara, Takumi Shiraishi, Takashi Ueda, Munehiro Ohashi, Osamu Ukimura, Kei Yamada

**Affiliations:** 1 Department of Diagnostic Radiology, Japanese Red Cross Kyoto Daini Hospital, Kyoto, Japan; 2 Department of Radiology, Kyoto Prefectural University of Medicine, Kyoto, Japan; 3 Department of Diagnostic Radiology, Japanese Red Cross Kyoto Daiichi Hospital, Kyoto, Japan; 4 The Clinical and Translational Research Center, University Hospital, Kyoto Prefectural University of Medicine, Kyoto, Japan; 5 Department of Urology, Kyoto Prefectural University of Medicine, Kyoto, Japan; 6 Department of Urology, Kyoto Tanabe Central Hospital, Kyotanabe, Japan

**Keywords:** Magnetic resonance imaging, Prostate cancer, Focal therapy, Cryotherapy, Microwave tissue coagulation therapy

## Abstract

**Purpose:**

This study compared the longitudinal changes in multiparametric magnetic resonance imaging (mpMRI) findings following lesion-targeted focal cryotherapy with those after microwave tissue coagulation (MTC) therapy for localized prostate cancer with the aim of determining their modality-specific imaging characteristics and evolution over time.

**Materials and methods:**

The study included 16 patients (17 procedures) who underwent cryotherapy and 33 patients (34 procedures) who received MTC therapy between March 2017 and February 2024. Serial mpMRI scans were retrospectively reviewed for treatment-induced signal changes on T1-weighted imaging, T2-weighted imaging, diffusion-weighted imaging, and dynamic contrast-enhanced magnetic resonance imaging (MRI). Three radiologists independently reviewed the images, and interobserver agreement was evaluated.

**Results:**

Early post-treatment MRI findings indicated distinct modality-specific patterns. Cryotherapy-treated lesions frequently demonstrated marked T1 hyperintensity, whereas MTC-treated lesions predominantly showed slight hyperintensity. On T2-weighted imaging and diffusion-weighted imaging, cryotherapy-treated lesions were characterized by hyperintensity with a hypointense rim, while MTC therapy was more likely to result in heterogeneous hypointensity. Early rim enhancement was common on dynamic contrast-enhanced MRI following cryotherapy (71.4%) and MTC (83.3%) and resolved by 23 and 41 months, respectively. In the late phase (> 12 months), imaging findings generally progressed toward fibrosis, which was characterized by hypointensity across all sequences without enhancement, although convergence timing varied from patient to patient.

**Conclusions:**

While there are distinct modality-specific differences in MRI characteristics in the early phase after between focal cryotherapy and MTC therapy for localized prostate cancer, late-stage findings converge, primarily reflecting fibrosis. These MRI features can help when monitoring the treatment response and guide appropriate follow-up planning.

## Introduction

Widespread adoption of prostate-specific antigen (PSA) testing and advances in imaging diagnostics and biopsy techniques have led to an increased rate of early detection of localized prostate cancer [1]. Whole-gland treatments such as radical prostatectomy and radiation therapy are widely used as standard therapeutic options [2, 3]. However, they are associated with a considerable risk of complications, including urinary incontinence, erectile dysfunction, and inability to ejaculate, which have a significant negative impact on patients’ quality of life [4, 5]. To mitigate these issues, focal therapy (FT) has been developed to selectively target the index lesion while preserving the surrounding normal tissue, thereby reducing treatment-related adverse events. While long-term data are limited, medium-term results for FT indicate effective cancer control with fewer complications [6–8].

Various FT modalities are available, including high-intensity focused ultrasound (HIFU), cryotherapy, focal laser ablation (FLA), and irreversible electroporation (IRE) [6]. More recently, microwave tissue coagulation (MTC) has been introduced as an additional option [9–11].

Given that residual normal prostate tissue secretes PSA regardless of the FT modality used, PSA alone is insufficient for detecting recurrence of cancer. Therefore, an international multidisciplinary consensus statement has recommended the use of multiparametric MRI (mpMRI) in conjunction with PSA measurement for post-treatment follow-up of FT [12]. Accurate interpretation of post-FT mpMRI is becoming increasingly important in the evaluation of treatment efficacy and optimization of patient management.

Although detailed reports on longitudinal MRI changes following each FT modality remain scarce, the existing research indicates that FT induces post-treatment changes and energy-specific alterations [13, 14]. A few studies have specifically described MRI findings after focal cryotherapy, highlighting characteristic early changes, such as T1 hyperintensity and rim enhancement, followed by progressive fibrosis and loss of enhancement in the late phase [15, 16]. However, no study has directly compared post-treatment MRI findings across the various FT modalities. Without such a comparison, it remains unclear whether follow-up strategies should be consistent across these modalities or tailored to each energy type. As FT is increasingly adopted, a precise understanding of modality-specific MRI changes is essential to ensure appropriate use of MRI in patient monitoring.

This study compared focal cryotherapy and focal MTC therapy when used to treat localized prostate cancer under standardized clinical conditions with the aim of identifying modality-specific MRI changes post-treatment and establishing a clearer understanding of their imaging characteristics.

## Materials and methods

### Patient selection and MRI follow-up

The study was approved by the Kyoto Prefectural University of Medicine Ethical Review Board (No. ERB-C-2220, 28 December 2021, and No. ERB-C-2966, 13 October 2023) and the Ethics Review Board of Kyoto Tanabe Central Hospital (No. 2023-009, 1 August 2023). The requirement for written informed consent was waived in view of the retrospective nature of the research and its use of clinical information without human biological samples. This waiver was granted in accordance with the Ethical Guidelines for Life Sciences and Medical Research Involving Human Subjects promulgated on 31 March 2021 and issued jointly by the Ministry of Education, Culture, Sports, Science and Technology; the Ministry of Health, Labour and Welfare; and the Ministry of Economy, Trade and Industry of Japan.

Focal cryotherapy was first introduced at our institution in March 2017 and remained the predominant FT modality until June 2021. In July 2021, a separate clinical trial protocol for cryotherapy was initiated, and all patients treated under that protocol were excluded from the present study. Focal MTC therapy was first performed at our institution in June 2019, and patients treated up to February 2024 were included in this study. The study included 16 patients who underwent focal cryotherapy and 33 who received focal MTC therapy at the University Hospital, Kyoto Prefectural University of Medicine or Kyoto Tanabe Central Hospital. These cohorts overlap with those included in our previous modality-specific studies [15, 17]. Both these lesion-targeted FT procedures are minimally invasive and were performed electively at each patient’s own expense. Both FTs were indicated for localized prostate cancer (cT2), confirmed by MRI–transrectal ultrasound (TRUS)-guided fusion biopsy. Patients who were deemed eligible for a minimally invasive procedure and had expressed a preference for FT were included in the study.

For safety reasons, tumors located within 5 mm of the membranous urethra were deemed unsuitable for focal cryotherapy [18], and tumors within 10 mm of the rectum were considered unsuitable for focal MTC therapy.

After treatment, both groups underwent follow-up MRI examinations. In the early phase of each treatment, additional MRI was performed at 3-month post-treatment to confirm safety, while standard follow-up was performed at 6-month intervals. A total of 110 MRI scans were performed after focal cryotherapy and 97 MRI scans were obtained after focal MTC therapy.

**Fig. 1:**
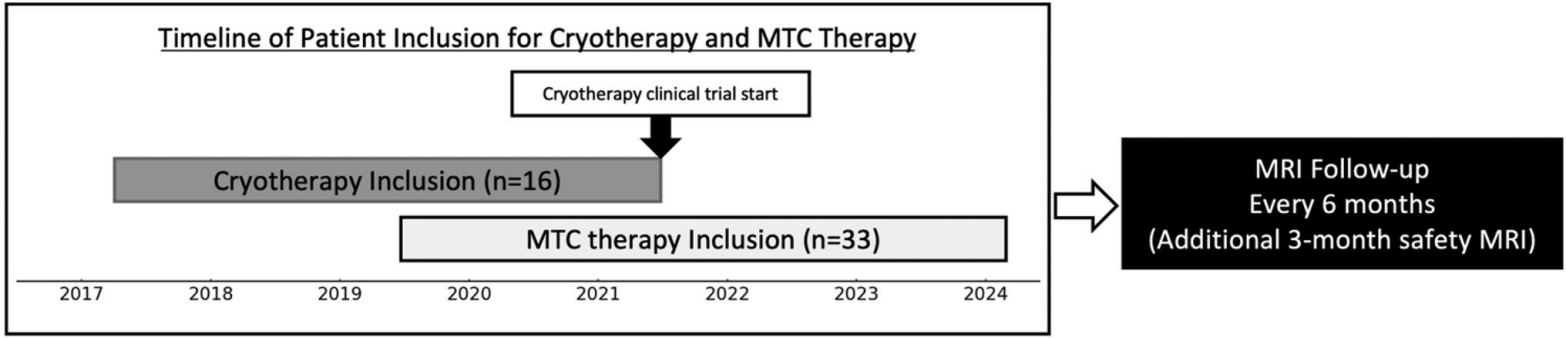
Diagram showing the patient selection and MRI follow-up process. This figure summarizes the timeline of patient enrolment and the schedule of post-treatment MRI follow-up in the present study. Focal cryotherapy was performed between March 2017 and June 2021 and focal MTC between June 2019 and February 2024. Patients who received cryotherapy and were enrolled in a separate clinical trial from July 2021 onward were excluded from this study. MRI follow-up was conducted at 3-month post-treatment to confirm early safety and subsequently at 6-month intervals. *MRI* magnetic resonance imaging, *MTC* microwave tissue coagulation

A summary of the patient selection and follow-up process is provided in Fig. 1.

### Imaging protocols

MpMRI was performed using a 1.5-T (Magnetom Altea, Siemens, Erlangen, Germany) or 3.0-T (Magnetom Skyra, Siemens, Erlangen, Germany or Ingenia Elition, Philips, Amsterdam, Netherlands) scanner with a phased array coil.

Axial T1-weighted imaging (T1WI), T2-weighted imaging (T2WI), diffusion-weighted imaging (DWI), and dynamic contrast-enhanced (DCE)–MRI were performed. The imaging protocols and parameters used were in accordance with the Prostate Imaging Reporting and Data System (PI-RADS) version 2.1 guideline [19]. Specific imaging parameters are shown in Table 1.

**Table 1:** Sequence parameters for the prostate magnetic resonance imaging protocol

3 T	Axial T1WI	Axial T2WI	Axial DWI	Axial DCE T1WI^a^
Pulse sequence	2D FSE	2D FSE	2D EPI	3D GRE
Fat saturation	−	−	+	+
Repetition time (ms)	590	5000	5100	4.2
Echo time (ms)	12	98	60	1.58
Matrix	256 × 256	640 × 640	160 × 160	512 × 512
Slice thickness (mm)	3	3	3.5	3
Interslice gap (mm)	0	0	0	0
Field of view (mm)	200	200	220	200
*b* value (s/mm^2^)			0, 1500	

1.5 T	Axial T1WI	Axial T2WI	Axial DWI	Axial DCE T1WI^a^
Pulse sequence	2D FSE	2D FSE	2D EPI	3D GRE
Fat saturation	−	−	+	+
Repetition time (ms)	500	3720	7510	3.9
Echo time (ms)	10	90	73	1.6
Matrix	288 × 288	288 × 288	220 × 220	256 × 256
Slice thickness (mm)	3	3	3	3
Interslice gap (mm)	0.3	0.3	0.3	0
Field of view (mm)	250	250	250	240
*b* value (s/mm^2^)			0, 1500	

*DCE* dynamic contrast-enhanced, *DWI* diffusion-weighted imaging, *EPI* echo planar imaging, *FSE* fast spin echo, *GRE* gradient echo, *T1WI* T1-weighted imaging, *T2WI* T2-weighted imaging

^a^Each DCE-T1WI was obtained after injection of 0.1 mmol/kg of meglumine gadoterate (Magnescope; Guerbet, Tokyo, Japan) at a rate of 2 mL/s

### Tumor ablation technique

All ablation procedures were performed under general anesthesia by the same urologist (OU). A transperineal approach was used with biplane (transverse and sagittal) real-time TRUS guidance and referenced to 3D cancer mapping using diagnostic MRI as well as the MRI–TRUS image fusion biopsy data set. For MRI–TRUS image fusion biopsy, the 3D MRI–TRUS fusion-based organ-tracking system (Urostation or Trinity, both from Koelis, Grenoble, France) was used to record and display the trajectories of both virtual and real biopsy procedures. This approach provided precise digital 3D positional data for MRI–visible lesions and cancer-positive biopsy tracks, ensuring accurate 3D cancer mapping as the treatment reference.

#### Cryotherapy

Cryotherapy was performed using a CryoHit machine (Boston Scientific, Marlborough, MA, USA). A urethral warming catheter circulated warm water to prevent urethral injury, and thermal sensors were placed at the Denonvilliers’ fascia, neurovascular bundle, external sphincter, and center of the tumor. Typically, three cryoprobes were inserted into the index lesion under real-time TRUS guidance using the biplane probe. As a reference, augmented reality software was used to visualize expansion from the cryoprobes in 3D, ensuring tumor coverage within lethal temperatures (below − 40 °C). Two or three freeze–thaw cycles were performed. Technical success was defined as the cancer lesion being under complete coverage within the lethal temperature zone (below − 40 °C), namely, over 5 mm inside from the edge of the ice ball (0 °C), with monitoring by real-time TRUS and multiple thermal sensors.

#### Microwave tissue coagulation

MTC was performed using the Microtaze-AFM-712 device (Alfresa Pharma Corporation, Osaka, Japan). Under real-time TRUS guidance using the biplane probe, the treatment needle was inserted into the target lesion, followed by insertions of additional needles into the perilesional area with reference to 3D cancer mapping using diagnostic MRI as well as the MRI–TRUS image fusion biopsy data set. A microwave output of 30 W was applied for 60 s per session. For complete ablation, multiple MTC sessions were performed to cover the target lesion with a 5-mm surrounding margin. Technical success was defined as complete disappearance of Doppler signals from the feeding vessels within the cancer lesion in comparison between intraoperative pre-ablation vs. post-ablation using Doppler function of real-time TRUS.

### Date acquisition and analysis

Pre-treatment and post-treatment diagnostic MRI findings were obtained for retrospective comparison of the MRI findings observed in the treated areas following cryotherapy and MTC therapy. Specifically, we evaluated signal changes in the treated area relative to the surrounding normal prostate tissue on T1WI, T2WI, and DWI, and the enhancement patterns of the treated area on DCE–MRI. Signal intensities and enhancement patterns were classified as follows.

*T1-weighted images*

(i) Marked hyperintensity, defined as a signal intensity equal to or greater than that of fatty tissue
(ii) Slight hyperintensity, defined as a signal intensity greater than that of normal prostate tissue but less than that of fatty tissue
(iii) Isointensity
(iv) Hypointensity

*T2-weighted images*

(i) Hyperintensity with a hypointense rim
(ii) Heterogeneous hypointensity with a hypointense rim
(iii) Hypointensity

*Diffusion-weighted images*

(i) Hyperintensity with a hypointense rim
(ii) Heterogeneous mixed signal intensity
(iii) Hypointensity

*Dynamic contrast-enhanced magnetic resonance images*

(i) Internal enhancement, defined as contrast enhancement within the treated area
(ii) Rim enhancement, defined as contrast enhancement along the boundary of the treated area
(iii) No enhancement

In addition to the above categories, linear T2 hyperintensity along the needle tract was recorded when observed.

**Fig. 2:**
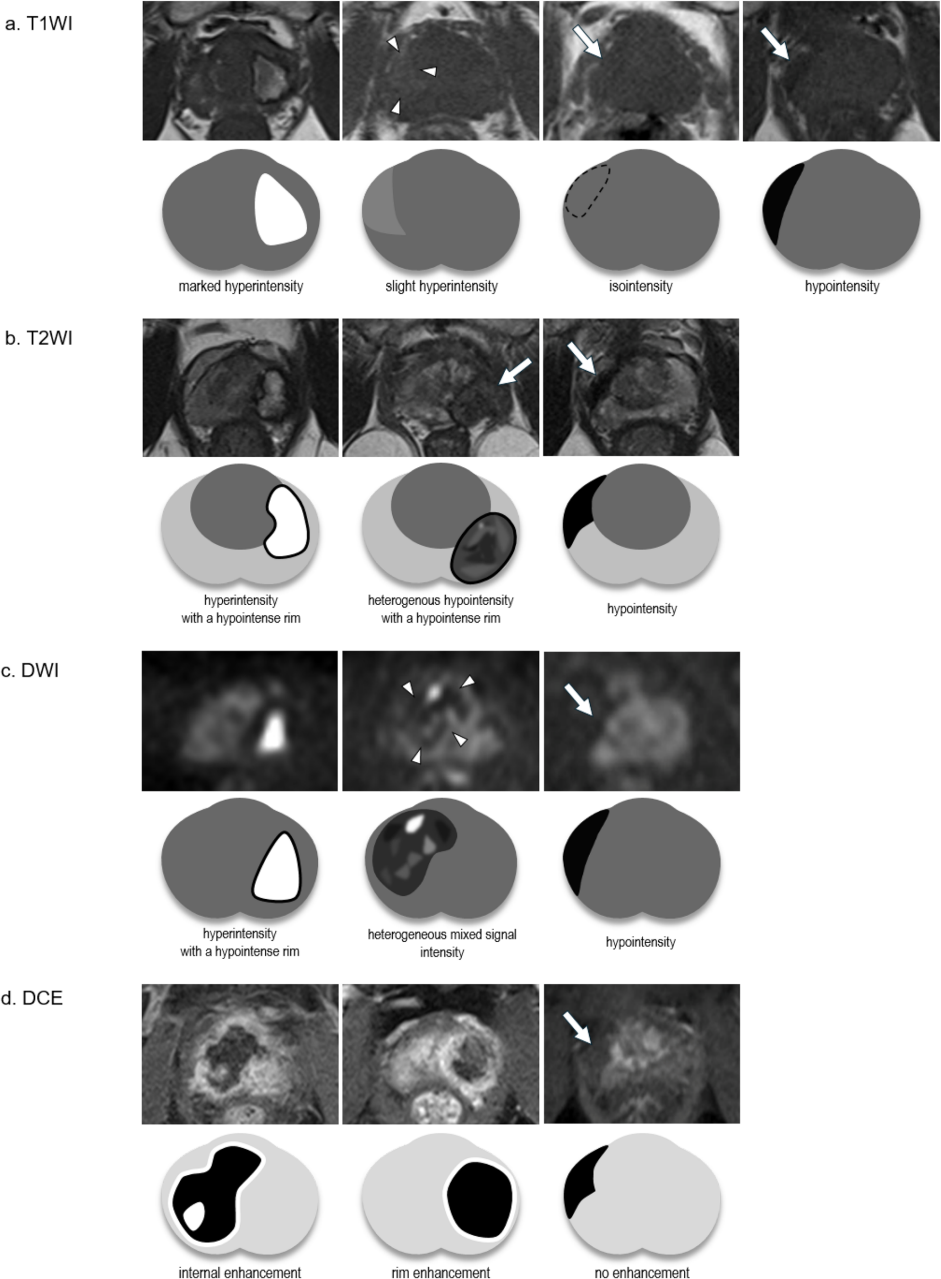
Examples of magnetic resonance imaging categorization and schemas. The categorization of signal changes and contrast enhancement patterns in the ablated area was as follows: **a** on T1WI, marked hyperintensity, slight hyperintensity, isointensity, or hypointensity; **b** on T2WI, hyperintensity with a hypointense rim, heterogeneous hypointensity with a hypointense rim, or hypointensity; **c** on DWI, hyperintensity with a hypointense rim, heterogeneous mixed signal intensity, or hypointensity; **d** on DCE–MRI, internal enhancement, rim enhancement, or no enhancement. *DCE–MRI* dynamic contrast-enhanced magnetic resonance imaging, *DWI* diffusion-weighted imaging, *T1WI* T1-weighted imaging, *T2WI* T2-weighted imaging

Examples of the classification criteria and schematic diagrams are provided in Fig. 2.

The classifications were performed by three radiologists working independently (BT, NK, and KY, with 7, 13, and 20 years of experience, respectively, in interpreting prostate MRI). The final classifications were determined by majority vote. Interobserver agreement was evaluated using the Fleiss kappa coefficient. The statistical analysis was performed using SPSS software version 29.0.0.0 (IBM Corp., Armonk, NY, USA).

## Results

### Patient data

Sixteen patients (17 procedures) who received cryotherapy and 33 patients (34 procedures) who received MTC therapy were included in the study. One patient in each group underwent repeat treatment for recurrence or residual disease.

The mean age was 68 years in both groups and the mean pre-treatment PSA level was 8.1 ng/mL for cryotherapy and 9.7 ng/mL for MTC therapy. The mean follow-up duration was 40 months for cryotherapy and 17 months for MTC therapy, reflecting the earlier introduction of cryotherapy at our institution. Sixteen patients in the cryotherapy group and 5 in the MTC group had follow-up data extending beyond 24 months.

The patients’ clinical characteristics, including the PI-RADS v2.1 categories of target lesions on pre-treatment MRI, are summarized in Table 2.

**Table 2:** Patient characteristics

Characteristic	Cryotherapy	MTC therapy
Patients, *n*	16	33
Procedures, *n*	17	34
Age, years	68 (51–81)	68 (50–85)
Pre-treatment PSA, ng/mL	8.1 (4.1–14.0)	9.7 (2.8–36.6)
PSA density, ng/mL/mL	0.36 (0.13–0.69)	0.34 (0.07–1.23)
PI-RADS v2.1 category		
3	2 (11.8)	11 (32.3)
4	14 (82.3)	19 (55.9)
5	1 (5.9)	4 (11.8)
Clinical T stage		
T2a	13 (81.25)	29 (87.9)
T2c	3 (18.75)	4 (12.1)
ISUP grade group		
1	0 (0)	4 (11.8)
2	7 (41.1)	11 (32.3)
3	10 (58.9)	11 (32.3)
4	0 (0)	8 (23.5)
Follow-up duration, months	40 (11–54)	17 (3–52)

Data are shown as the mean (range) or as the number (percentage) as appropriate

*ISUP* International Society of Urological Pathology, *MTC* microwave tissue coagulation, *PI-RADS* Prostate Imaging Reporting and Data System, *PSA* prostate-specific antigen

### MRI signal changes and contrast enhancement patterns

On average, follow-up MRI was performed at 3, 6, 12, 17, 23, 29, 35, 41, 45, and 51 months after treatment. The number of patients who underwent MRI at each timepoint is shown in Table 3 for cryotherapy and in Table 4 for MTC therapy.

**Table 3:** Summary of magnetic resonance imaging signal changes and contrast enhancement patterns following focal cryotherapy

Mean interval since cryotherapy, months (range)	3 (1–4) *n* = 7	6 (5–8) *n* = 15	12 (9–14) *n* = 17	17 (15–20) *n* = 13	23 (20–26) *n* = 13	29 (26–31) *n* = 13	35 (33–37) *n* = 12	41 (38–43) *n* = 9	45 (44–47) *n* = 7	51 (49–54) *n* = 4
T1WI										
Marked hyperintensity	6/7 (85.7%)	1/15 (6.7%)	–	–	–	–	–	–	–	–
Slight hyperintensity	–	8/15 (53.3%)	3/17 (17.6%)	1/13 (7.7%)	–	–	–	–	–	–
Isointensity	–	3/15 (20.0%)	6/17 (35.3%)	1/13 (7.7%)	–	–	–	–	–	–
Hypointensity	1/7 (14.3%)	3/15 (20.0%)	8/17 (47.1%)	11/13 (84.6%)	13/13 (100%)	13/13 (100%)	12/12 (100%)	9/9 (100%)	7/7 (100%)	4/4 (100%)
T2WI										
Hyperintensity with a hypointense rim	4/7 (57.1%)	2/15 (13.3%)	–	–	–	–	–	–	–	–
Heterogeneous hypointensity with a hypointense rim	2/7 (28.6%)	4/15 (26.7%)	1/17 (5.9%)	–	–	–	–	–	–	–
Hypointensity	1/7 (14.3%)	9/15 (60.0%)	16/17 (94.1%)	13/13 (100%)	13/13 (100%)	13/13 (100%)	12/12 (100%)	9/9 (100%)	7/7 (100%)	4/4 (100%)
DWI										
Hyperintensity with a hypointense rim	5/7 (71.4%)	1/15 (6.7%)–	–	–	–	–	–	–	–	–
Heterogeneous mixed signal intensity	1/7 (14.3%)	–	–	–	–	–	–	–	–	–
Hypointensity	1/7 (14.3%)	14/15 (93.3%)	17/17 (100%)	13/13 (100%)	13/13 (100%)	13/13 (100%)	12/12 (100%)	9/9 (100%)	7/7 (100%)	4/4 (100%)
DCE–MRI										
Internal enhancement	1/7 (14.3%)	–	–	–	–	–	–	–	–	–
Rim enhancement	5/7 (71.4%)	4/14 (28.6%)	1/15 (6.7%)	1/11 (9.1%)	–	–	–	–	–	–
No enhancement	1/7 (14.3%)	10/14 (71.4%)	14/15 (93.3%)	10/11 (90.9%)	11/11 (100%)	11/11 (100%)	10/10 (100%)	8/8 (100%)	7/7 (100%)	3/3 (100%)

*DCE* dynamic contrast-enhanced, *DWI* diffusion-weighted imaging, *MRI* magnetic resonance imaging, *T1WI* T1-weighted imaging, *T2WI* T2-weighted imaging

**Table 4:** Summary of magnetic resonance imaging signal changes and contrast enhancement patterns following focal MTC therapy

Mean interval since MTC therapy, months (range)	3 (1–4) *n* = 13	6 (5–8) *n* = 28	12 (9–14) *n* = 19	17 (15–20) *n* = 13	23 (20–26) *n* = 10	29 (26–31) *n* = 5	35 (33–37) *n* = 3	41 (38–43) *n* = 2	45 (44–47) *n* = 2	51 (49–54) *n* = 2
T1WI										
Marked hyperintensity	1/13 (7.7%)	–	–	–	–	–	–	–	–	–
Slight hyperintensity	7/13 (53.8%)	9/28 (32.1%)	3/19 (15.8%)	–	–	–	–	–	–	–
Isointensity	3/13 (23.1%)	12/28 (42.9%)	7/19 (36.8%)	2/13 (15.4%)	1/10 (10%)	–	–	–	–	–
Hypointensity	2/13 (15.4%)	7/28 (25%)	9/19 (47.4%)	11/13 (84.6%)	9/10 (90%)	5/5 (100%)	3/3 (100%)	2/2 (100%)	2/2 (100%)	2/2 (100%)
T2WI										
Hyperintensity with a hypointense rim	2/13 (15.4%)	2/28 (7.1%)	–	–	–	–	–	–	–	–
Heterogeneous hypointensity with a hypointense rim	8/13 (61.5%)	11/28 (39.3%)	3/19 (15.8%)	–	–	–	–	–	–	–
Hypointensity	3/13 (23.1%)	15/28 (53.6%)	16/19 (84.2%)	13/13 (100%)	10/10 (100%)	5/5 (100%)	3/3 (100%)	2/2 (100%)	2/2 (100%)	2/2 (100%)
DWI										
Hyperintensity with a hypointense rim	2/13 (15.4%)	–	–	–	–	–	–	–	–	–
Heterogeneous mixed signal intensity	8/13 (61.5%)	10/28 (35.7%)	2/19 (10.5%)	1/13 (7.7%)	–	–	–	–	–	–
Hypointensity	3/13 (23.1%)	18/28 (64.3%)	17/19 (89.5%)	12/13 (92.3%)	10/10 (100%)	5/5 (100%)	3/3 (100%)	2/2 (100%)	2/2 (100%)	2/2 (100%)
DCE–MRI										
Internal enhancement	–	–	–	–	–	–	–	–	–	–
Rim enhancement	10/12 (83.3%)	13/25 (52%)	5/17 (29.4%)	2/12 (16.7%)	–	1/5 (20%)	1/3 (33.3%)	–	–	–
No enhancement	2/12 (16.7%)	12/25 (48%)	12/17 (70.6%)	10/12 (83.3%)	10/10 (100%)	4/5 (80%)	2/3 (66.7%)	2/2 (100%)	2/2 (100%)	2/2 (100%)

*DCE* dynamic contrast enhancement, *DWI* diffusion-weighted imaging, *MRI* magnetic resonance imaging, *MTC* microwave tissue coagulation, *T1WI* T1-weighted imaging, *T2WI* T2-weighted imaging

Interobserver agreement for the interpretation of MRI sequences ranged from moderate to almost perfect, with the lowest agreement observed for T1WI (*κ* = 0.549) and the highest for DWI (*κ* = 0.809). The kappa coefficients for each sequence are shown in Table 5.

**Table 5:** Interobserver agreement on magnetic resonance imaging categories

MRI sequence	Kappa value (95% CI)
T1WI	0.549 (0.495–0.602)
T2WI	0.699 (0.635–0.763)
DWI	0.809 (0.744–0.875)
DCE–MRI	0.794 (0.714–0.873)

*CI* confidence interval, *DCE* dynamic contrast enhancement, *DWI* diffusion-weighted imaging, *MRI* magnetic resonance imaging, *T1WI* T1-weighted imaging, *T2WI* T2-weighted imaging

**Fig. 3:**
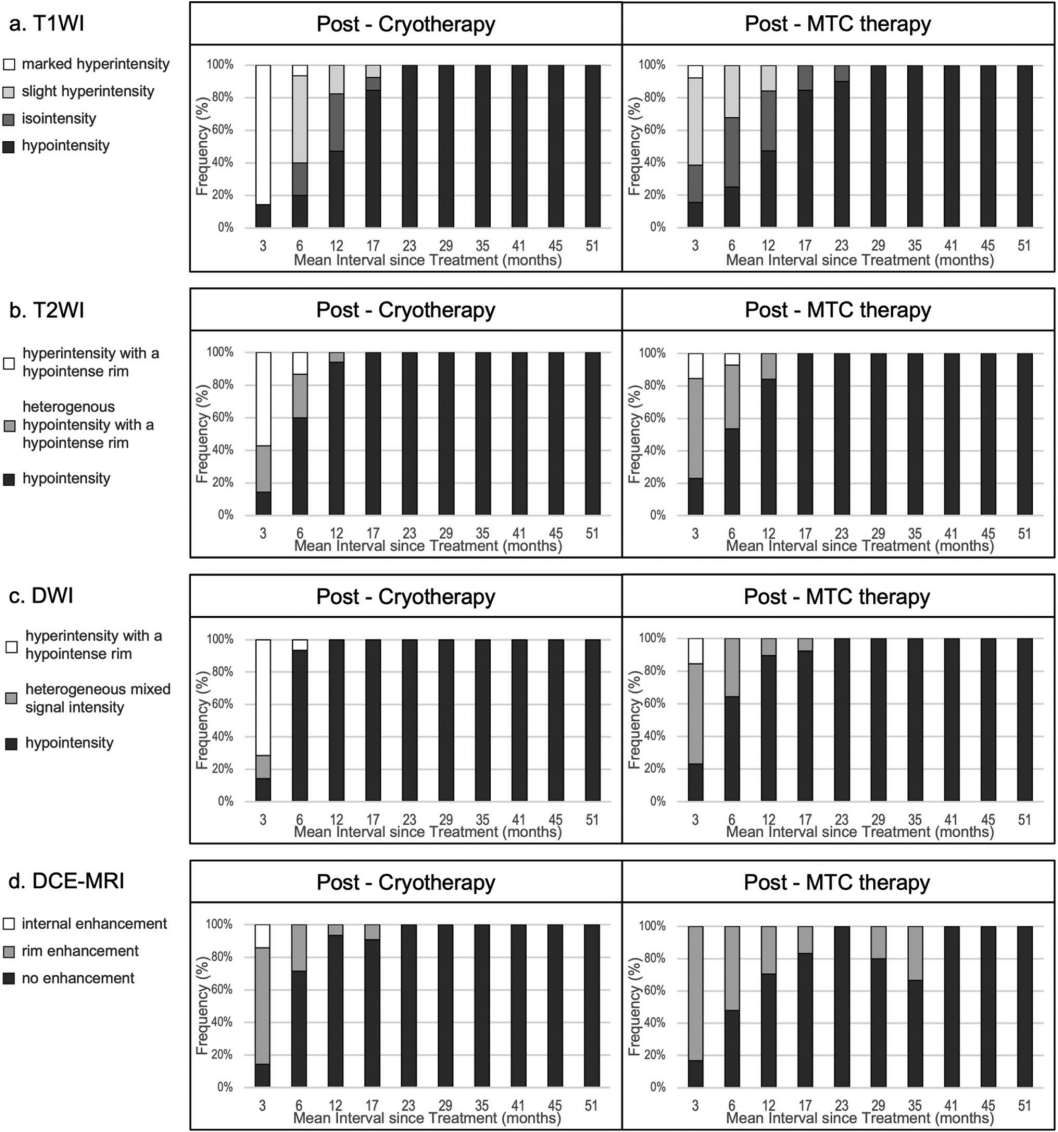
Frequency of signal changes on magnetic resonance images after focal cryotherapy and microwave tissue coagulation therapy. Bar graphs showing the frequency of MRI signal changes, contrast enhancement patterns for each sequence, with a mean follow-up duration of 3–51 months after both therapies. *DCE–MRI* dynamic contrast-enhanced magnetic resonance imaging; *DWI* diffusion-weighted imaging, *MRI* magnetic resonance imaging, *MTC* microwave tissue coagulation, *T1WI* T1-weighted imaging, *T2WI* T2-weighted imaging

In both study groups, the first post-treatment MRI scans revealed signal changes in all target lesions, along with disappearance of findings suggestive of cancer. Tables 3 and 4 summarize the changes in signal intensity and contrast enhancement patterns for the target lesions, with Table 3 corresponding to cryotherapy and Table 4 to MTC therapy. The distribution of MRI findings for each sequence is shown in bar graph form in Fig. 3.

In the early post-treatment period, marked hyperintensity on T1WI was more frequently observed in the cryotherapy group, whereas slight hyperintensity was more common in the MTC therapy group. On T2WI and DWI scans, hyperintensity with a hypointense rim was seen more often in the cryotherapy group and heterogeneous hypointensity or mixed signal was observed more frequently in the MTC therapy group. These patterns gradually diminished over time, transitioning to hypointensity. At 23 months after cryotherapy and 29 months after MTC therapy, all patients showed hypointensity across all non-contrast-enhanced sequences.

On contrast-enhanced imaging, rim enhancement was initially observed in 71.4% of patients in the cryotherapy group and in 83.3% of those in the MTC therapy group at 3-month post-treatment. Over time, this enhancement disappeared in both groups. No enhancement was detected in any case at 23 months in the cryotherapy group or at 41 months in the MTC therapy group. In contrast, internal enhancement was rare, observed in only one patient in the cryotherapy group at 3 months with disappearance by 6 months.

When fibrosis was defined as the appearance of low signal intensity on all non-contrast sequences (T1WI, T2WI, and DWI) together with the absence of enhancement on DCE–MRI, such imaging changes were confirmed in 16 patients in the cryotherapy group and 14 in the MTC group. The median time to convergence was 16.5 months (range 5.8–25.3) for cryotherapy and 14.5 months (range 3.9–27.8) for MTC therapy.

Transient linear T2 hyperintensity along the needle tract was frequently observed on the first post-treatment MRI obtained following MTC therapy but was not seen after cryotherapy. This finding decreased over time and was no longer observed after 17 months.

**Fig. 4:**
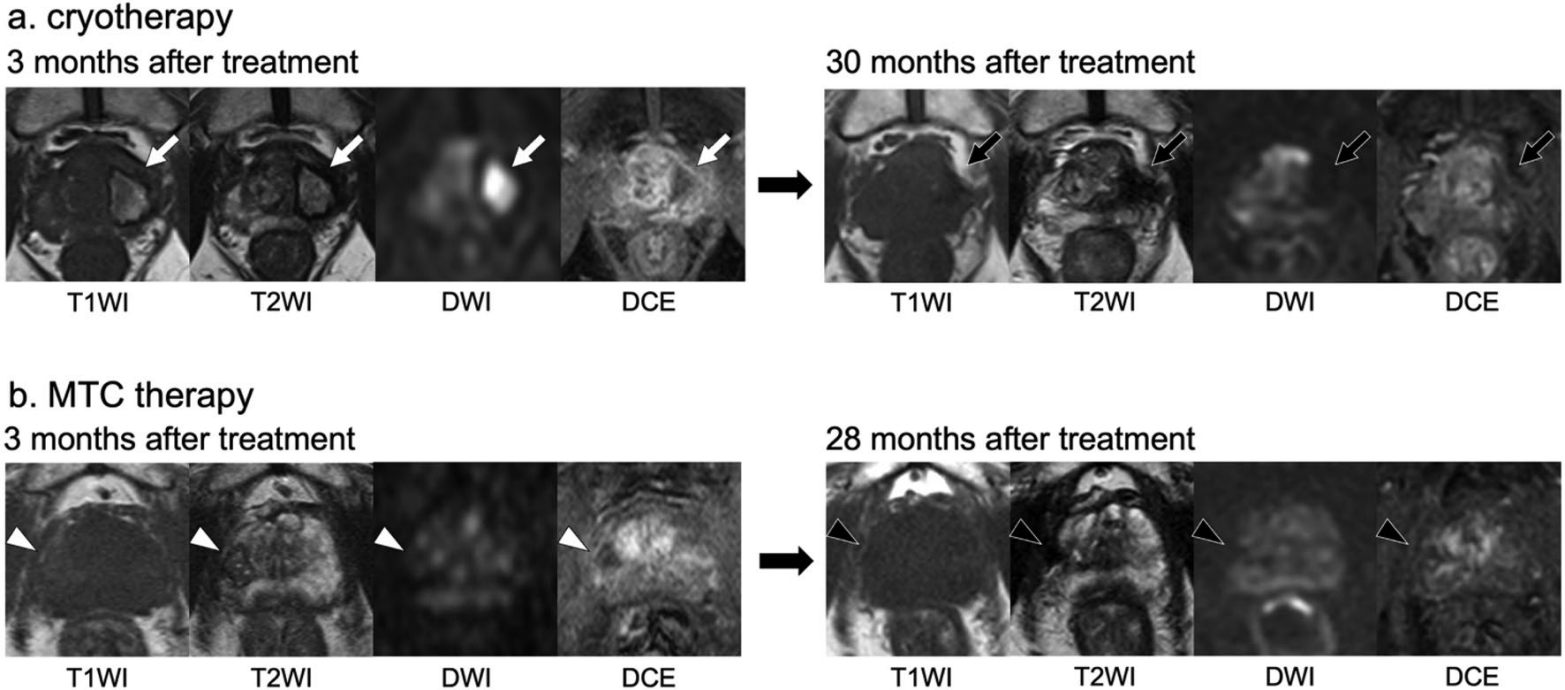
Typical temporal changes in findings on magnetic resonance images after focal cryotherapy and microwave tissue coagulation therapy. **a** At 3 months after cryotherapy, T1WI showed marked hyperintensity, while T2WI and DWI showed hyperintensity with a hypointense rim. DCE–MRI demonstrated rim enhancement. By 30 months, T1WI, T2WI, and DWI showed hypointensity, and DCE–MRI showed no enhancement. **b** At 3 months after MTC therapy, T1WI showed slight hyperintensity, T2WI showed heterogeneous hypointensity with a hypointense rim, and DWI showed heterogeneous mixed signal intensity. DCE–MRI showed rim enhancement. A small spot of high T2 signal within the treated area corresponds to the needle tract. By 28 months, T1WI, T2WI, and DWI showed hypointensity, and DCE–MRI showed no enhancement. Although early post-treatment MRI findings differed between the two therapies, late-stage findings were similar. *DCE–MRI* dynamic contrast-enhanced magnetic resonance imaging, *DWI* diffusion-weighted imaging, *MTC* microwave tissue coagulation, *T1WI* T1-weighted imaging, *T2WI* T2-weighted imaging

Figure 4 shows the typical MRI findings in two cases, one after cryotherapy and one after MTC therapy.

## Discussion

We have previously reported longitudinal MRI findings following focal cryotherapy and MTC therapy as modality-specific studies [15, 17]. Building on these investigations, the present study represents a direct comparison of MRI findings following cryotherapy and MTC therapy under standardized conditions over an extended period. The early post-treatment MRI findings (i.e., up to and including 12 months after therapy in this study) differed noticeably between cryotherapy and MTC therapy, while the late-stage findings (i.e., beyond 12 months) indicated common signal changes. Although both modalities demonstrated similar contrast enhancement patterns after treatment, the progression of these changes over time differed to some extent. These findings highlight both modality-specific and common MRI changes, as well as their chronological progression.

Cryotherapy is the second most widely used FT modality for prostate cancer worldwide after HIFU [6]. It is well established for prostate cancer and has been extensively studied in other organs, including the kidney and liver. In contrast, MTC has been widely used for liver tumors but has only recently been applied for prostate cancer, with our hospital playing a pioneering role in its clinical application [11]. The emerging use of MTC therapy provides a unique opportunity to explore its potential as a novel FT modality for localized prostate cancer.

Clinically, cryotherapy and MTC therapy have complementary actions, allowing for strategic use based on tumor location. Cryotherapy is contraindicated for tumors near the membranous urethra in view of the risk of injury to the external urethral sphincter muscle [18], whereas MTC therapy can be safely applied near the membranous urethra owing to its well-defined treatment boundary. Conversely, MTC therapy is unsuitable for tumors near the rectum because of the high risk of thermal damage [11]. However, once cryotherapy has started and the cryoprobe is fixed, the prostate and probes can be manually lifted away from the rectum to avoid injury—a technique known as the “prostate-lifted-up technique”, originally developed by Dr. Ukimura [20]. Selecting the appropriate lesion-targeted focal ablation therapy based on tumor location may improve the efficacy of treatment while reducing complications.

The differences in MRI findings between the two modalities can be attributed to their distinct underlying mechanisms. Cryotherapy disrupts cell membranes by forming ice crystals and inducing osmotic stress [21], which leads to marked hemorrhage and a strong inflammatory response that accelerates tissue repair [22, 23]. These biological effects are reflected in the early post-treatment MRI findings: marked hyperintensity on T1WI and hyperintensity on both T2WI and DWI, consistent with hemorrhage [15] and similar to findings in rabbit kidney models [24]. In contrast, MTC induces cell death as a result of protein denaturation caused by rapid heating and thermal fixation [25], which preserves cellular structures while causing limited inflammation and a different tissue repair profile [26, 27]. The slight hyperintensity on T1WI, heterogeneous hypointensity on T2WI, and mixed signal intensity on DWI in the early post-treatment period after MTC therapy likely correspond to varying degrees of necrosis [17], as reported in liver studies [28–30].

Linear T2 hyperintensity along the needle tract was observed only after MTC therapy and during the early post-treatment period. Pathological studies have consistently demonstrated tissue loss surrounding the needle tract after MTC therapy, which appears as hyperintense areas on T2WI [11, 31, 32]. Although this finding has no pathological significance, it serves as a useful marker for objectively identifying the needle placement site on MRI after treatment.

Rim enhancement was observed on DCE–MRI in the early post-treatment period and gradually disappeared over time; this feature was common after both treatments in this study. Rim enhancement has been attributed to formation of granulation tissue, an inflammatory response, and hyperemia at the periphery of the ablation zone [15–17]. In this study, internal enhancement on DCE–MRI was observed in only one case following cryotherapy. In renal cell carcinoma, post-cryotherapy enhancement has been reported to persist for up to 9 months, potentially reflecting residual vasculature within the ablation zone, transient inflammation, or tissue repair [33, 34]. A similar phenomenon may occur in the prostate, but further investigation is required.

In this study, both cryotherapy and MTC therapy resulted in T2WI hypointensity and loss of contrast enhancement in the late post-treatment period, consistent with post-treatment scar formation. This fibrosis-related endpoint was further quantified in the present study and found to have a similar median time to convergence for both treatments.

**Fig. 5:**
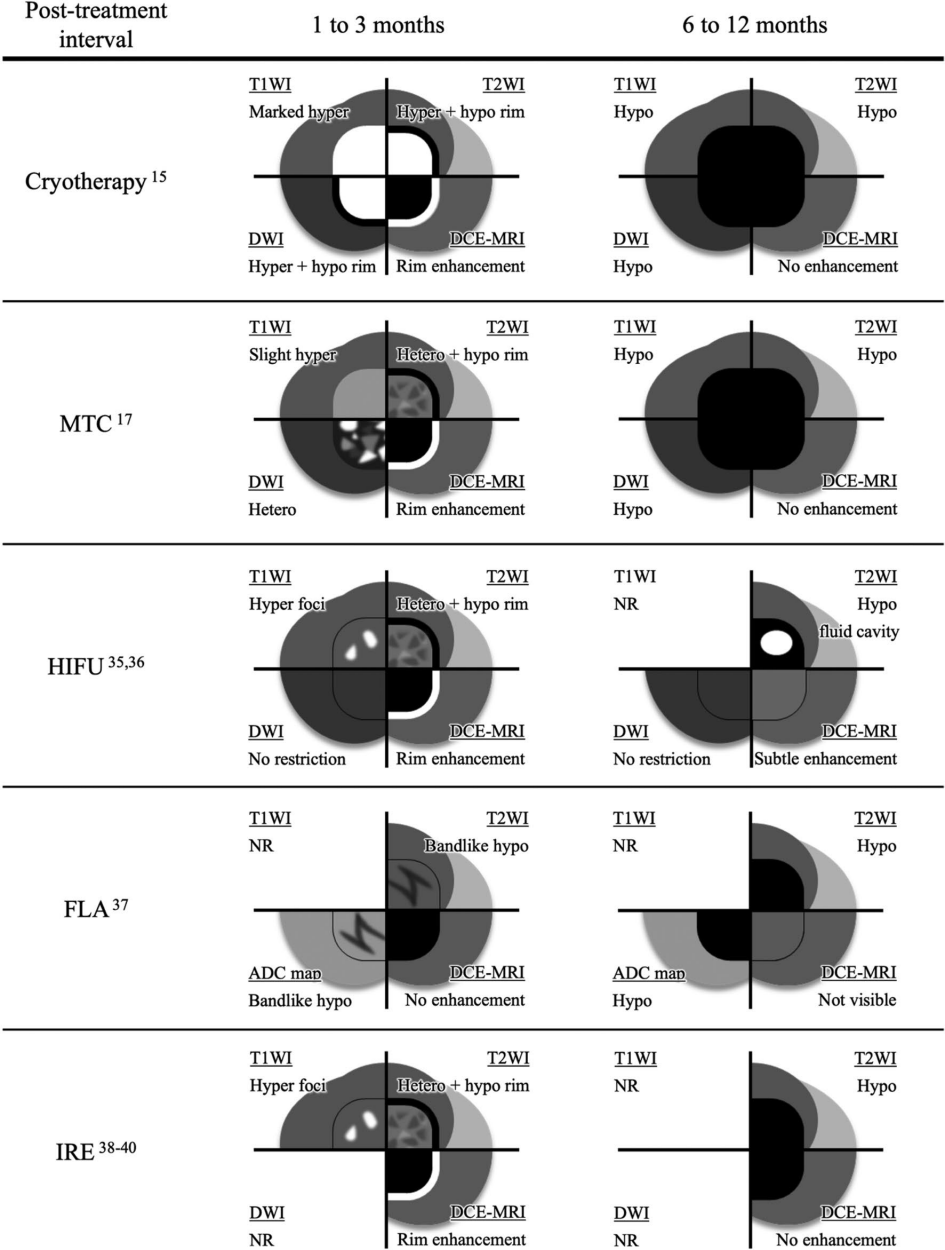
Timeline showing typical multiparametric magnetic resonance imaging findings according to focal therapy modality for prostate cancer. This schematic diagram presents representative mpMRI features at 1–3 months and 6–12 months after the following five focal therapy modalities: HIFU, FLA, IRE, cryotherapy, and MTC. Imaging findings are shown across four MRI sequences: T1WI, T2WI, DWI, and DCE–MRI. The summary is based on previously published studies and our institutional experience with cryotherapy and MTC and aims to facilitate cross-modality comparison and clinical interpretation of post-treatment MRI changes. *ADC* apparent diffusion coefficient, *DCE* dynamic contrast enhancement, *DWI* diffusion-weighted imaging, *FLA* focal laser ablation, *HIFU* high-intensity focused ultrasound, *IRE* irreversible electroporation, *MRI* magnetic resonance imaging, *MTC* microwave tissue coagulation, *NR* not reported, *T1WI* T1-weighted imaging, *T2WI* T2-weighted imaging

The findings of this study are consistent with previous reports indicating that FT induces both general post-treatment changes and energy-specific effects [13, 14]. To facilitate cross-modality comparison, representative mpMRI findings at 1–3 months and 6–12 months after each FT modality, including cryotherapy, MTC, HIFU, FLA, and IRE, are schematically summarized in Fig. 5 [15, 17, 35–40]. At 1–3-month post-treatment, mpMRI findings varied markedly according to treatment modality, likely reflecting differences in the extent of necrosis, inflammation, and hemorrhage. Rim enhancement, which was observed after all therapies except FLA, likely reflects common tissue responses similar to those seen after cryotherapy and MTC [38, 41, 42]. At 6–12-month post-treatment, MRI findings often included T2 hypointensity across all FTs, consistent with treatment-induced fibrosis. In HIFU-treated cases, persistent subtle enhancement associated with chronic inflammation and fluid cavities communicating with the urethra has been reported [36]. In FLA-treated lesions at 12 months, ablation sites were mostly undetectable on DCE–MRI, with enhancement similar to that of the surrounding peripheral zone [37].

The findings of this study clarify the differences in post-treatment MRI findings between cryotherapy and MTC therapy and may provide a reference for clinical evaluation. For example, following cryotherapy, marked hemorrhagic changes are often observed at 3-month post-treatment, and transient internal enhancement may also be present. These features could make it difficult to differentiate normal post-treatment changes from a residual or recurrent tumor, suggesting that assessment may become more reliable at approximately 6 months. In contrast, MTC therapy is less likely to show significant hemorrhagic changes or internal enhancement at 3 months. However, at this timepoint, DWI frequently shows a mixed signal pattern. By 6 months, hypointensity patterns become more predominant, yet mixed signal patterns can still be observed. In such instances, distinguishing small areas of high signal from residual tumor may be challenging, and careful interpretation is required.

This study has several limitations. First, it was performed retrospectively and included limited sample sizes, particularly for patients who received cryotherapy and those who underwent long-term follow-up after MTC therapy, where only a few patients were followed beyond 2 years. In the MTC group, patient numbers decreased over time, with < 50% undergoing MRI at the latest follow-up points. This limited long-term follow-up should be considered when interpreting the longitudinal imaging findings. However, the imaging findings generally stabilized in the late post-treatment period as a consequence of scar formation. Second, the study did not include a direct correlation of pathological results with imaging findings. Given that FT is a minimally invasive approach, routine pathological validation is not feasible in clinical practice. Prostatectomy for confirmation is not a viable option, making histopathological assumptions reliant on previously reported animal studies. Third, this study did not assess tumor recurrence either within the treated field (infield) or in untreated areas (outfield). However, the primary aim was to characterize the normal evolution of imaging findings after FT. Future research should investigate the MRI characteristics indicative of recurrence infield and those suggestive of a new lesion outfield following FT.

In conclusion, this research provides a direct comparison of MRI findings following cryotherapy with those after MTC therapy for localized prostate cancer. While early post-treatment MRI findings differed markedly between the two treatments, both ultimately led to similar fibrosis-related changes in the late phase. These insights should improve the interpretation of post-treatment MRI findings, help to optimize patient management, and may contribute to the development of standardized MRI follow-up protocols for FT.
